# Computational Modeling Reduces Leaflet Modification Rates in Valve-In-Valve and Redo Transcatheter Aortic Valve Replacement

**DOI:** 10.1016/j.jacadv.2026.103377

**Published:** 2026-10-23

**Authors:** Nicholas J. Valle, Omar A. Saleh, Omar Jafar, Juan Munoz-Moreno, Ann M. Harper, Chelsea M. Martin, Joshua D. Dolsen, Raymond L. Benza, Clinton D. Kemp, Parth K. Parikh, Paul D. Mahoney, Deepak R. Talreja, Benjamin D. Peterson, Joshua A. Cohen, Matthew R. Summers

**Affiliations:** a Department of Internal Medicine, Macon and Joan Brock Virginia Health Sciences at Old Dominion University, Eastern Virginia Medical School, Norfolk, Virginia, USA; b Macon and Joan Brock Virginia Health Sciences at Old Dominion University, Eastern Virginia Medical School, Norfolk, Virginia, USA; c Enterprise Analytics, Sentara Health, Virginia Beach, Virginia, USA; d Department of Cardiac Quality and Compliance, Sentara Medical Group, Sentara Health, Virginia Beach, Virginia, USA; e Department of Cardiology, Macon and Joan Brock Virginia Health Sciences at Old Dominion University, Eastern Virginia Medical School, Norfolk, Virginia, USA; f Department of Cardiothoracic Surgery, Sentara Medical Group, Sentara Health, Virginia Beach, Virginia, USA; g Department of Structural Cardiology, Sentara Medical Group, Sentara Health, Virginia Beach, Virginia, USA; h Department of Structural Cardiology, Eastern Carolina University Medical Center, Greenville, North Carolina, USA; i Department of Interventional Cardiology, Intermountain Medical Center, Intermountain Health, Salt Lake City, Utah, USA

**Keywords:** computational predictive modeling, coronary obstruction, leaflet modification, preprocedural planning, transcatheter aortic valve replacement, valve-in-valve

## Abstract

**Background:**

Coronary obstruction (CO) complicates valve-in-valve (ViV) and redo transcatheter aortic valve replacement (TAVR) at higher rates than native TAVR. Leaflet modification mitigates this risk but increases procedural burden. Traditional computed tomography angiography (CTA)-based risk stratification relies on static parameters that may overestimate risk; computational predictive modeling (CPM) uses patient-specific data to refine risk assessment.

**Objectives:**

The study aims to characterize leaflet modification rates in conventional ViV TAVR and redo-TAVR and evaluate the impact of CPM preprocedural planning augmentation on leaflet modification rates.

**Methods:**

A total of 157 consecutive ViV TAVR or redo-TAVR patients treated from 2022 to 2025 were retrospectively analyzed. CO risk status was derived from traditional CTA parameters. Leaflet modification rates among CTA high-risk subgroups (non-CPM, n = 62; and CPM, n = 29) were compared using Boschloo's one-sided exact test. CPM risk-reclassification patterns were studied using counterfactual binomial analysis.

**Results:**

Leaflet modification occurred in 26.3% of ViV TAVR and 24.1% of redo-TAVR procedures that used only conventional CTA-based planning methods. Among patients deemed high risk for CO by conventional methods, leaflet modification was significantly lower in the CPM-augmented subgroup vs that in the non-CPM subgroup (24.1% vs 46.8%; OR: 2.76; 95% CI: 1.03-7.40; *P* = 0.021). CPM-augmented planning reclassified 82.8% of patients to low-risk status, and 79.2% of reclassified patients were managed without leaflet modification. No COs occurred during the study period.

**Conclusions:**

Among ViV TAVR and redo-TAVR patients at high risk of CO based on conventional CTA-based planning, the use of CPM to augment standard risk assessment was associated with a significant reduction in leaflet modification procedures.

Transcatheter aortic valve replacement (TAVR) has transformed the management of aortic stenosis in recent decades. Valve-in-valve (ViV; TAVR-in-surgical aortic valve replacment) and redo-TAVR (TAVR-in-TAVR) are established options for patients who develop restenosis of prior surgical and transcatheter aortic valve (AV) bioprostheses. In ViV patients, rates of coronary obstruction (CO) during valve implantation are higher than those in native TAVR (2%-4% vs <1%, respectively), with in-hospital mortality exceeding 40% when CO occurs.^1^, ^2^, ^3^, ^4^ Leaflet modification (LM) procedures such as bioprosthetic native aortic scallop intentional laceration to prevent iatrogenic coronary artery obstruction (BASILICA) are used to mitigate CO risk, but these adjuncts substantially increase procedural complexity, duration, contrast exposure, and radiation time.^5^, ^6^ Although BASILICA has demonstrated durable procedural effectiveness in preventing CO, the key unresolved question is not whether LM works, but how to better identify which patients truly need it.^7^, ^8^

CO risk stratification has remained largely stagnant despite growing TAVR volume and patient complexity. Standard assessment relies on coronary height (CH), virtual valve-to-coronary distance (VTc), and virtual valve-to-sinotubular junction distance (VTstj) which are static 2D measurements derived from manual implantation of simplified virtual transcatheter heart valve (THV) models into computed tomography angiography (CTA) renderings.^1^, ^9^, ^10^, ^11^, ^12^, ^13^ Risk predictions using these methods are often conservative, potentially resulting in the overuse of LM procedures. Computational predictive modeling (CPM) systems are now being used to address this limitation.^12^ The technology leverages patient-specific CTA data to generate high-fidelity 3D virtual models of postdeployment THVs and aortic anatomy. In addition to enhancing visual assessment of existing anatomy and predicted anatomical outcomes, measurements such as the distance from the virtual valve leaflet to coronary ostium relative to ostial diameter (DLC/d) can be derived from these virtual 3D models.^14^, ^15^, ^16^ The clinical impact of this technology on LM rates, particularly among ViV TAVR and redo-TAVR patients, remains largely undefined.

We retrospectively analyzed rates of LM procedures in consecutive patients undergoing ViV TAVR or redo-TAVR at a single high-volume center over a 4-year period. We evaluated whether CPM-augmented preprocedural planning safely reduced LM rates among patients deemed high risk of CO by conventional CTA, hypothesizing that CPM-augmented planning would yield a reduction in LM procedures despite comparable CTA-derived risk profiles.

## Methods

### Study design and population

This study was a retrospective analysis of consecutive patients undergoing ViV TAVR or redo-TAVR at a single high-volume structural heart center between January 1, 2022, and December 31, 2025. The study population included all patients older than 18 years with a prior surgical or transcatheter aortic bioprosthesis. Exclusion criteria included native TAVR procedures and incomplete data. No patients were excluded on the basis of low CTA quality; all studies were sufficient to derive the parameters of interest (ie, CH, VTc, and VTstj). Furthermore, image quality and CPM findings were reviewed by a high-volume multidisciplinary heart team consisting of at least 1 structural cardiologist, 1 structural imaging cardiologist, 1 cardiothoracic surgeon, and 1 to 2 advanced practice providers. CPM cases also had at least 1 company affiliated engineer involved in output derivation and validation. Preprocedural planning and clinical decision-making were multidisciplinary in all cases. Clinical decisions were made after consideration of all relevant patient history, clinical data (including CPM where applicable), and procedural expectations. All procedures were performed by high-volume interventional cardiologists.

Patients were stratified into subgroups based on CO risk status (high vs low risk) using traditional CTA parameters (ie, CH, VTc, and VTstj) and standard multidisciplinary heart team adjudication. CTA parameters were derived by manual implantation of virtual THV models into CTA renderings using TeraRecon (Aquarius) software, which was used consistently in all cases. Patients were considered high risk for CO if one or more parameters fell below institutional thresholds (CH <10 mm, VTc <4 mm, and VTstj <2 mm). Patients with patent coronary bypass grafts supplying otherwise high-risk coronary arteries were considered low risk. Patients with incomplete CTA data were adjudicated using a blinded chart review or direct remeasurement.

Patients who underwent CPM in addition to the standard preprocedural planning workflow were identified (CPM group, n = 29). All other patients were managed with traditional CTA-based planning alone (non-CPM group, n = 128). Rates of LM procedures among each subgroup were recorded. All CPM outputs included in the study were produced prior to the index ViV TAVR or redo-TAVR. Retrospective CPM after the initiation of this study was not feasible, as it would have required clinically unnecessary testing. Therefore, we retrospectively evaluated physician decision-making patterns and clinical outcomes among patients who had CPM in the past. No part of the present study was prospective.

### Data collection and endpoints

Data were extracted from the institutional Society of Thoracic Surgeons/ACC TVT (American College of Cardiology Transcatheter Valve Therapy) registry, electronic medical record, and CPM company records. Baseline characteristics, preprocedural planning parameters, procedural data, and in-hospital and short-term outcomes data were collected. The primary endpoint was the rate of adjunctive LM procedures (ie, BASILICA or other techniques) in the computed tomography (CT) high-risk subgroup stratified by CPM-augmented planning status. Secondary endpoints included rates of CO, rates of LM in non-CPM ViV TAVR and redo-TAVR patients, contrast exposure, fluoroscopy time, procedure time, and short-term major adverse cardiovascular events (MACE), defined as the composite of all-cause death, ischemic stroke, and myocardial infarction during the index admission and at 30 days, 3 months, and 6 months. Myocardial infarction and bleeding were defined by Valve Academic Research Consortium version 2 criteria.

### Preprocedural planning workflows

The non-CPM cohort was managed solely with traditional CTA-based preprocedural planning. The CPM cohort received traditional CTA-based planning in addition to CPM evaluation. Among this group, patient-specific CTA data were processed by the CPM company (DASI Simulations, LLC) to generate high-fidelity virtual 3D models of postdeployment anatomy which included the in situ bioprosthesis, proposed index THV(s), aortic root, and calcium. Structures are differentiated by color (Figure 1). Modeling was conducted off site, and output reports were provided by the company to the heart team 1 to 2 days after the initial request. The primary CPM-derived CO risk-prediction parameter was the DLC/d ratio. A DLC/d ≤0.7 was considered high risk for CO. The algorithm used by the CPM platform for virtual model production is proprietary and was not made available to clinicians. The developer maintains that they use a physics-based platform which features a proprietary finite-element analysis system to generate predictive THV expansion models into CTA renderings creating a 3D model. Reported performance of the platform is further delineated in the PrecisionTAVI 501(k) summary [K223809]. The platform is currently Food and Drug Administration (FDA) cleared for CPM in TAVR and does not yet have formal FDA clearance for ViV TAVR and redo-TAVR specifically. Further details about the modeling platform are included in Supplemental Table 1, which is formatted to mirror a checklist table for emerging computational modeling technologies proposed by Kagiyama et al (Supplemental Table 1).^17^ We aim to report our single-center experience using this CPM platform, not provide additional verification and validation data. Clinicians should use caution when generalizing our results to other populations, and clarity surrounding model verification and validation remains an important issue as CPM and related technologies are becoming increasingly common in clinical practices.

**Figure 1:**
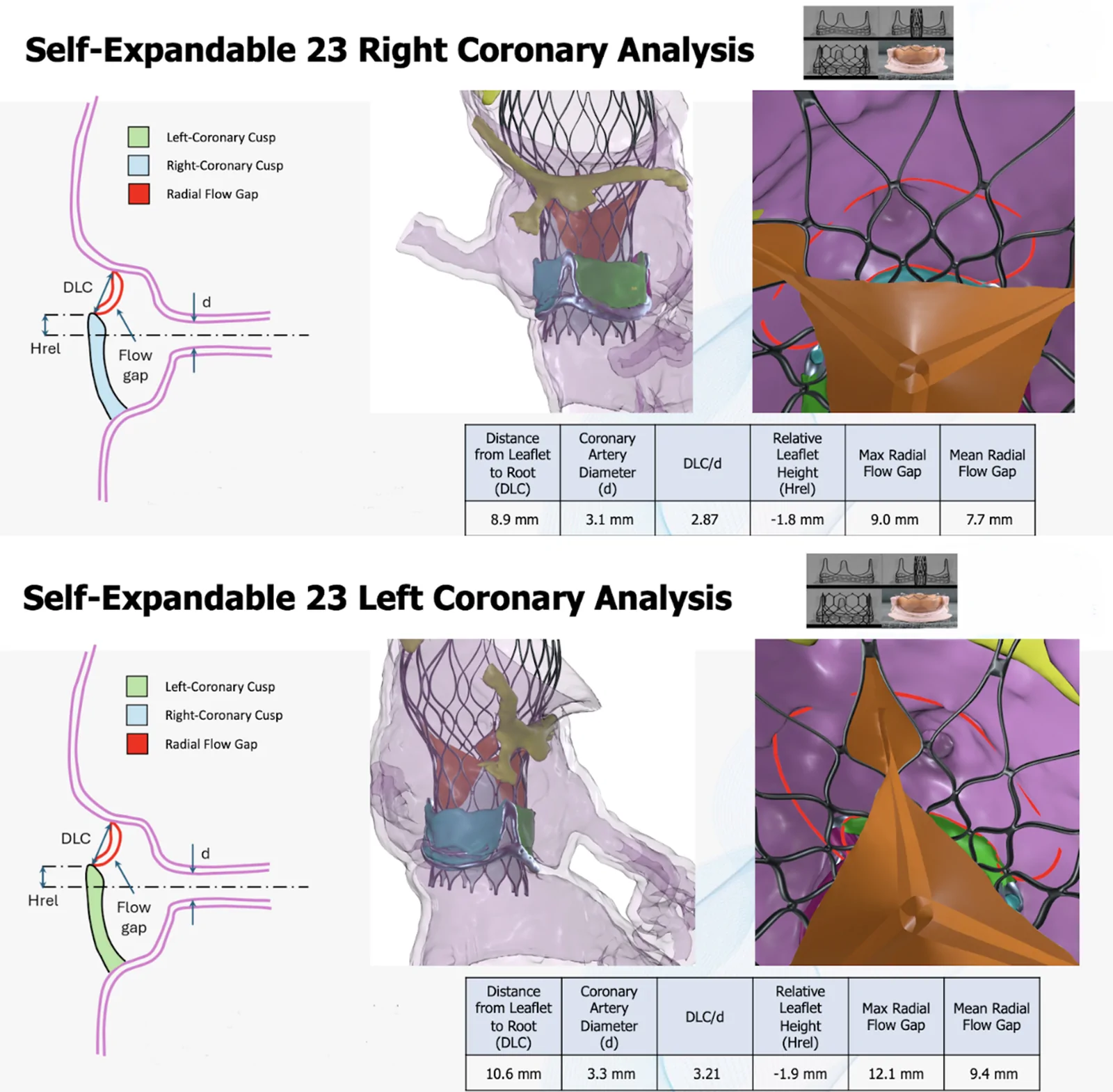
Diagram of Computational Modeling Report Sample component of the computational predictive modeling reports used in the study. The model is depicting a simulated valve-in-valve transcatheter aortic valve replacement with a virtual self-expanding valve model inside the existing surgical bioprosthesis. Surgical bioprosthetic leaflets are color-coded to designate coronary and noncoronary cusps. The patient’s anatomy is highlighted in purple. Calcium is displayed in yellow.

The way this CPM model is used to assess CO risk in ViV TAVR and redo-TAVR is conceptually identical to that in native TAVR. The coronary ostial diameter (d) is derived directly from the patient’s CTA and is not influenced by the presence of pre-existing bioprostheses. The distance from the virtual THV leaflet to the coronary ostium (DLC) is derived by measuring the distance from the virtual THV leaflet to the CTA-derived anatomical reconstruction. The decision to perform LM remained at the discretion of the multidisciplinary team in all cases and was in no case driven by CPM outputs alone. CPM became available at our center in January 2024. From that point forward, nearly all ViV TAVR and redo-TAVR cases were referred for CPM unless conventional CTA preprocedural planning findings were such that the patient’s CO risk profile was unambiguous (ie, there was a clear indication for or against LM). In this way, the CPM group was largely clinician selected based on multidisciplinary risk assessment.

### Statistical analysis

Continuous variables are presented as mean ± SD and compared using independent samples *t*-tests. Categorical variables are presented as counts and percentages and compared using Fisher's exact test unless otherwise specified. The primary comparison of LM rates in the CTA high-risk subgroup was performed using a one-sided Boschloo's exact test. Odds ratios with 95% Wilson confidence intervals are reported.

CPM risk-reclassification patterns within the CPM group are presented as a proportion. Among CTA high-risk patients reclassified to low risk by CPM, a one-sided binomial test was used to evaluate for a significant reduction in LM procedures compared to the counterfactual reference. All other analyses used two-sided tests with a significance threshold of *P* < 0.05. No formal sample size calculation was performed given the retrospective exploratory nature of the study. All analyses were performed using Python (version 3.11) with SciPy, NumPy, and statsmodels libraries.

### Ethical considerations

This study was approved by our center’s Institutional Review Board with an approved waiver of informed consent to perform a retrospective chart review of past events. No new CPM reports were generated for the purposes of this study. Patients receiving CPM were made aware that the results would contribute to clinical decision-making. All procedures were conducted in accordance with the Declaration of Helsinki and applicable institutional guidelines.

## Results

### Study population and baseline characteristics

A total of 157 patients undergoing ViV TAVR or redo-TAVR between January 1, 2022, and December 31, 2025, were included (Figure 2). Of these, 29 patients received CO risk planning augmentation with CPM. One hundred twenty-eight patients were managed using only traditional CTA parameters (non-CPM). Baseline characteristics are summarized in Table 1.

**Figure 2:**
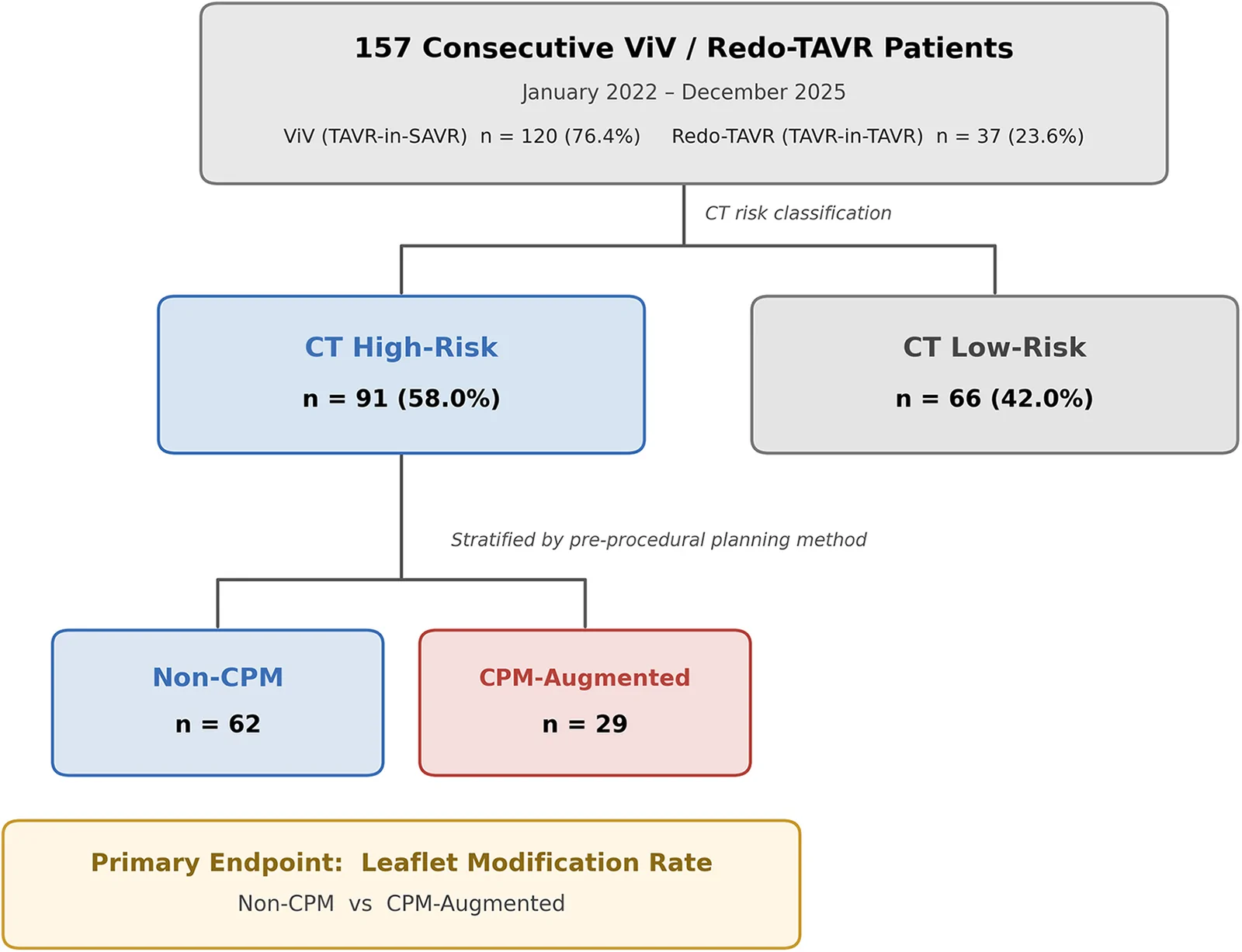
Diagram of Study Design Patients undergoing ViV TAVR or redo-TAVR were stratified by CT-based coronary obstruction risk classification. CT high-risk patients were further stratified by preprocedural planning method: non-CPM (n = 62) vs CPM (n = 29). The primary analysis compared leaflet modification rates between CT high-risk subgroups. Statistical tests are summarized in the lower panel. CPM = computational predictive modeling; CT = computed tomography; TAVR = transcatheter aortic valve replacement; ViV = valve-in-valve.

**Table 1:** Baseline Characteristics of Patients Undergoing ViV TAVR or Redo-TAVR (N = 157)

	Non-CPM (n = 128)	CPM (n = 29)	*P* Value
Demographics			
Age, y	72.2 ± 11.3	71.8 ± 12.9	0.867
Female, n (%)	46/128 (35.9%)	18/29 (62.1%)	**0.012**
BMI, kg/m^2^	29.0 ± 6.9	31.4 ± 7.6	0.125
Race			
White, n (%)	105/128 (82.0%)	20/29 (69.0%)	
Black/African American, n (%)	21/128 (16.4%)	8/29 (27.6%)	
Asian, n (%)	1/128 (0.8%)	1/29 (3.4%)	
Comorbidities			
Diabetes mellitus, n (%)	52/128 (40.6%)	12/29 (41.4%)	1.000
Hypertension, n (%)	115/128 (89.8%)	28/29 (96.6%)	0.469
Dialysis, n (%)	6/128 (4.7%)	2/29 (6.9%)	0.641
Chronic lung disease, n (%)	33/128 (25.8%)	10/29 (34.5%)	0.361
Liver disease, n (%)	8/128 (6.2%)	1/29 (3.4%)	1.000
PAD, n (%)	21/128 (16.4%)	4/29 (13.8%)	1.000
Atrial fibrillation, n (%)	67/128 (52.3%)	18/29 (62.1%)	0.411
Atrial flutter, n (%)	37/128 (28.9%)	9/29 (31.0%)	0.824
Carotid stenosis, n (%)	8/128 (6.2%)	0/29 (0.0%)	0.353
Prior CVA, n (%)	79/128 (61.7%)	20/29 (69.0%)	0.528
Heart failure, n (%)	104/128 (81.2%)	26/29 (89.7%)	0.415
Prior MI, n (%)	24/128 (18.8%)	6/29 (20.7%)	0.797
Surgical history			
Prior CABG, n (%)	38/128 (29.7%)	6/29 (20.7%)	0.371
Prior PCI, n (%)	26/128 (20.3%)	7/29 (24.1%)	0.622
Prior pacemaker, n (%)	18/128 (14.1%)	3/29 (10.3%)	0.767
Prior aortic valve procedure, n (%)	128/128 (100.0%)	29/29 (100.0%)	1.000
Clinical characteristics			
STS risk score, %	5.5 ± 5.2	6.1 ± 5.1	0.583
KCCQ overall summary	47.2 ± 25.9	40.0 ± 24.7	0.180
LVEF, %	51.9 ± 14.4	54.8 ± 16.0	0.374
AV annulus area, mm^2^	405.8 ± 103.2	348.4 ± 90.4	**0.010**
AV annulus perimeter, mm	72.4 ± 10.3	62.8 ± 15.0	**0.009**
AV area, cm^2^	0.9 ± 0.5	0.8 ± 0.3	0.112
AV mean gradient, mm Hg	42.2 ± 20.1	40.4 ± 15.5	0.602
AV peak gradient, mm Hg	69.4 ± 32.0	67.7 ± 22.8	0.739
AV peak velocity, m/s	4.0 ± 1.0	4.1 ± 0.7	0.617
AV annular calcification, n (%)	6/128 (4.7%)	6/29 (20.7%)	**0.010**
In situ AV prosthesis			
Surgical aortic valve	99/128 (77.3%)	21/29 (72.4%)	
Balloon-expandable valve	15/128 (11.7%)	5/29 (17.2%)	
Self-expanding valve	14/128 (10.9%)	3/29 (10.3%)	
Valve-in-valve as index procedure	99/128 (77.3%)	21/29 (72.4%)	0.629
Redo-TAVR as index procedure	29/128 (22.7%)	8/29 (27.6%)	

*P* values are derived from chi-squared or Fisher’s exact test (categorical) or independent *t*-test (continuous). **Bold***P* values represent *P* < 0.05.

AV = aortic valve; BMI = body mass index; CABG = coronary artery bypass graft; CPM = computational predictive modeling; CVA = cardiovascular accident; KCCQ = Kansas City Cardiomyopathy Questionnaire; LVEF = left ventricular ejection fraction; MI = myocardial infarction; PAD = peripheral arterial disease; PCI = percutaneous coronary intervention; STS = Society of Thoracic Surgeons; TAVR = transcatheter aortic valve replacement; ViV = valve-in-valve.

The cohorts were well matched across most demographic, clinical, and comorbidity characteristics. The average age between groups was similar: 72.2 ± 11.3 years in non-CPM vs 71.8 ± 12.9 years in the CPM cohort (*P* = 0.87). Society of Thoracic Surgeons predicted risk of mortality (5.5% ± 5.2% vs 6.1% ± 5.1%; *P* = 0.58), Kansas City Cardiomyopathy Questionnaire overall summary score (47.2 ± 25.9 vs 40.0 ± 24.7; *P* = 0.18), and left ventricular ejection fraction (51.9% ± 14.4% vs 54.8% ± 16.0%; *P* = 0.37) were also comparable between cohorts (non-CPM vs CPM, respectively). Rates of prior cardiac procedures and AV hemodynamic parameters did not differ significantly.

Female patients represented a greater proportion of the CPM cohort (62.1%) when compared with the non-CPM cohort (35.9%; *P* = 0.02). Patients in the CPM cohort also had a significantly smaller AV annular area (348.4 ± 90.4 vs 405.8 ± 103.2 mm^2^, respectively; *P* = 0.01) and annular valve perimeter (62.8 ± 15.0 vs 72.4 ± 10.3 mm, respectively; *P* < 0.001) than the non-CPM cohort. Aortic annular calcification was more prevalent in the CPM cohort (20.7% vs 4.7%, respectively; *P* = 0.010).

### Coronary obstruction risk profile

CTA-based CO risk assessment outcomes were captured for 29 of 29 (100%) CPM patients and 128 of 128 (100%) non-CPM patients. All 29 CPM patients (100%) were classified as high risk for CO by traditional methods compared with 62 of 128 non-CPM patients (48.4%; *P* < 0.0001) (Table 2).

**Table 2:** Leaflet Modification Rates Among CT High-Risk Patients and CPM Reclassification Patterns

	Non-CPM (n = 128)	CPM-Augmented (n = 29)	*P* Value
CT high risk, n (%)	62/128 (48.4%)	29/29 (100.0%)	<0.001
Left coronary height, mm	11.3 ± 5.2	11.7 ± 4.0	0.760
Right coronary height, mm	12.8 ± 6.0	11.8 ± 5.2	0.541
Left VTc, mm	5.7 ± 2.6	4.7 ± 2.4	0.162
Right VTc, mm	5.4 ± 2.6	4.5 ± 2.8	0.288
CT high-risk subgroup	n = 62	n = 29	
LM in CT high risk, n (%)	29/62 (46.8%)	7/29 (24.1%)	0.021
CPM reclassification			
CT high risk → CPM low risk (downgraded), n (%)	—	24/29 (82.8%)	
Downgraded → no LM, n (%)	—	19/24 (79.2%)	0.008
Coronary obstruction events, n	0	0	

All 29 CPM-augmented patients were classified as high risk by CT and/or multidisciplinary heart team review (100% vs 48.4%, *P* < 0.0001). In the CT high-risk subgroup, CPM-augmented patients had a LM rate of 24.1% vs 46.8% in non-CPM patients (*P* = 0.021).

CPM = computational predictive modeling; CT = computed tomography; LM = leaflet modification; VTc = valve-to-coronary distance.

### Baseline LM rates and procedure types

In the overall study population (n = 157), ViV TAVR was performed in 120 patients (76.4%) and redo-TAVR in 37 patients (23.6%). Across the total study population (n = 157), adjunctive LM was performed in 40 patients (25.5%). BASILICA was the predominant technique (33 of 40; 82.5%), followed by Picardia Shortcut (5 of 40; 12.5%) and unspecified leaflet splitting techniques (2 of 40; 5.0%) (Table 3). Among both the non-CPM and CPM cohorts, the most common pre-existing bioprosthetic valve type was surgical (77.3% vs 72.4%, respectively; *P* = 0.629). Self-expanding valve (SEV) use during the index procedure (ie, ViV TAVR or redo-TAVR) was numerically higher in the CPM cohort (25 of 29; 86.2% vs 90 of 128; 70.3%, respectively; *P* = 0.104).

**Table 3:** Leaflet Modification Procedures Among Non-CPM and CPM-Augmented Cohorts

Outcome	Non-CPM	CPM-Augmented
Any leaflet modification	33/128 (25.8%)	7/29 (24.1%)
BASILICA	31/128	2/29
Picardia	2/128	3/29
Unspecified	0/128	2/29
Fisher’s exact *P* value	1.0000	
Odds ratio (95% CI)	1.09	

Counts include BASILICA and all other leaflet modification techniques.

BASILICA = bioprosthetic native aortic scallop intentional laceration to prevent iatrogenic coronary artery obstruction; CPM = computational predictive modeling.

### LM rates in the non-CPM and CPM subgroups

Rates of LM among non-CPM ViV TAVR and redo-TAVR patients are reported separately: LM was performed in 26 of 99 non-CPM ViV TAVR patients (26.3%) and 7 of 29 non-CPM redo-TAVR patients (24.1%) (Figure 3, Table 4). Among the non-CPM cohort, CT high-risk classification was the principal driver of LM procedures: Twenty-nine of 62 CT high-risk patients (46.8%) underwent LM compared with 4 of 66 CT low-risk patients (6.1%) (Table 2). On case-level review, the 4 CT low-risk patients who underwent LM did so on the basis of preprocedural planning considerations that were not initially captured by our methods (ie, using solely CH, VTc, and VTstj). For example, in 3 of these cases, the coronary risk plane (ie, the vertical relationship between the top of the pre-existing bioprosthesis to the coronary ostium) was nonfavorable, leading the heart team to pursue LM despite reassuring traditional CTA parameters. In the fourth case, the patient required bioprosthesis fracture prior to ViV TAVR, which led to an expected reduction of an already borderline VTc (4.5-3.5 mm).

**Figure 3:**
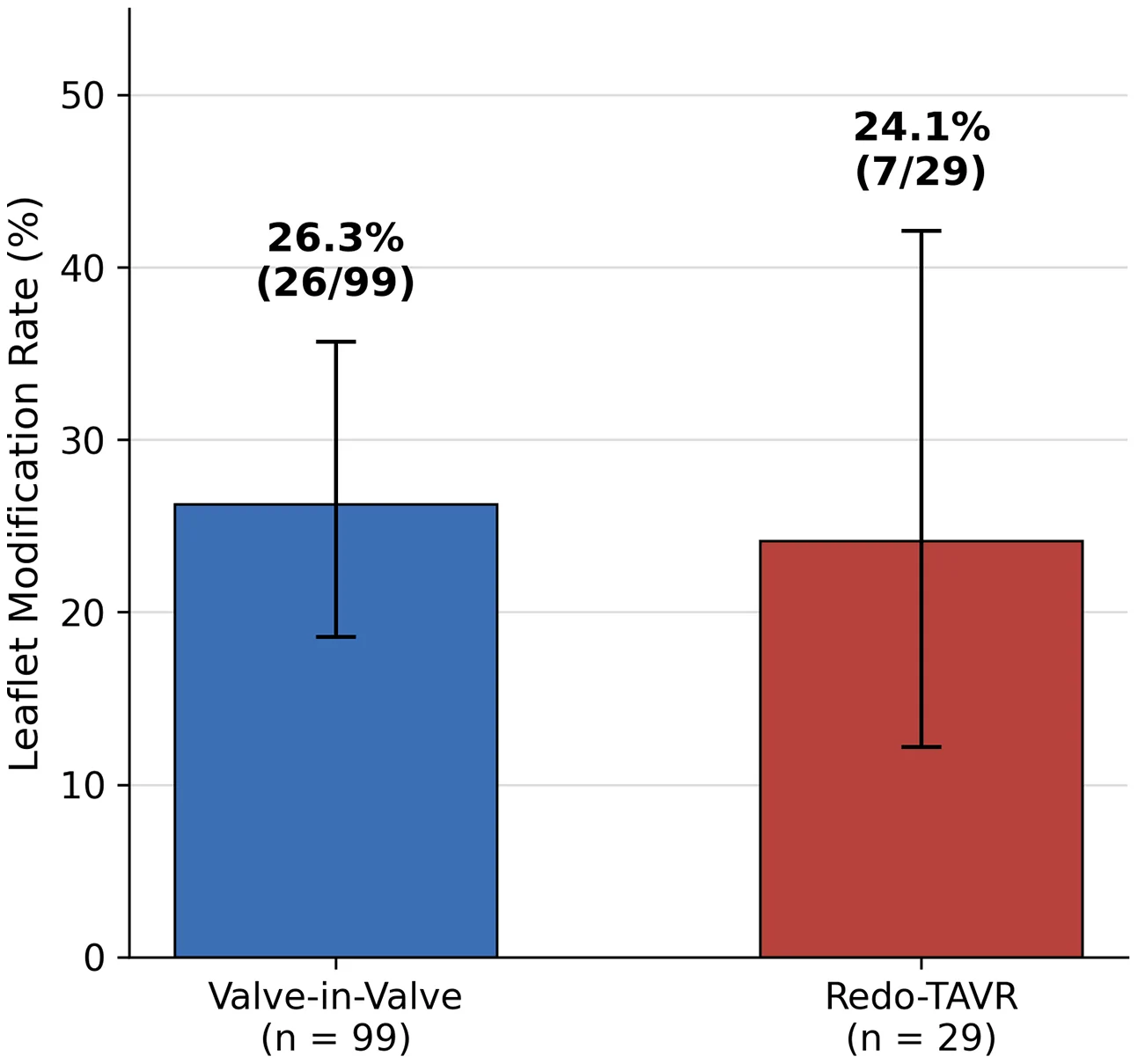
Leaflet Modification Rates by Procedure Type: Non-CPM Cohort Leaflet modification occurred in 26 of 99 (26.3%) valve-in-valve TAVR procedures and 7 of 29 (24.1%) redo procedures. CPM = computational predictive modeling; TAVR = transcatheter aortic valve replacement.

**Table 4:** Leaflet Modification Rates by Procedure Type in the Conventional Planning (Non-CPM) Cohort

Procedure Type	N	LM (n)	LM Rate
ViV	99	26	26.3%
Redo	29	7	24.1%

ViV = valve-in-valve; other abbreviations as in Table 2.

### LM rates stratified by CTA-based risk status

Without stratification by CTA-based risk status, rates of LM were similar between the total non-CPM and CPM cohorts: 33 of 128 (25.8%) vs 7 of 29 (24.1%), respectively (*P* = 1.000; OR: 1.09) (Table 3, Figure 4A). The primary analysis was then restricted to only those patients meeting traditional CTA high-risk criteria: 62 of 128 (48.4%) non-CPM patients and all 29 (100%) CPM patients (Table 2, Figure 4B). Among these risk-matched cohorts, LM rates were significantly lower among CPM patients: Seven of 29 CPM (24.1%) patients received LM compared with 29 of 62 (46.8%) non-CPM patients (OR: 2.76; 95% CI: 1.03-7.40; Boschloo's exact one-sided *P* = 0.021). Among the 29 CPM patients, 24 (82.8%) were downgraded from high risk to low risk following CPM-augmented planning, while 5 (17.2%) remained classified as high risk (Table 2, Central Illustration). Among the 24 risk-downgraded patients, 19 (79.2%) were ultimately managed without LM. The observed LM rate in the risk-downgraded group (5 of 24, 20.8%) was also significantly lower than the high-risk non-CPM reference rate of 46.8% (one-sided binomial *P* = 0.008(Table 2, Figure 4C). Five risk-reclassified patients underwent LM despite reassuring CPM findings. On case-level review, these patients had borderline conventional CTA findings (ie, bilateral low VTc values or nonreassuring coronary risk planes) that, in our early institutional experience with CPM planning, prompted the multidisciplinary heart team to proceed with LM out of an abundance of caution. No CO events occurred in any patient reclassified to low risk via CPM-augmented planning.

**Figure 4:**
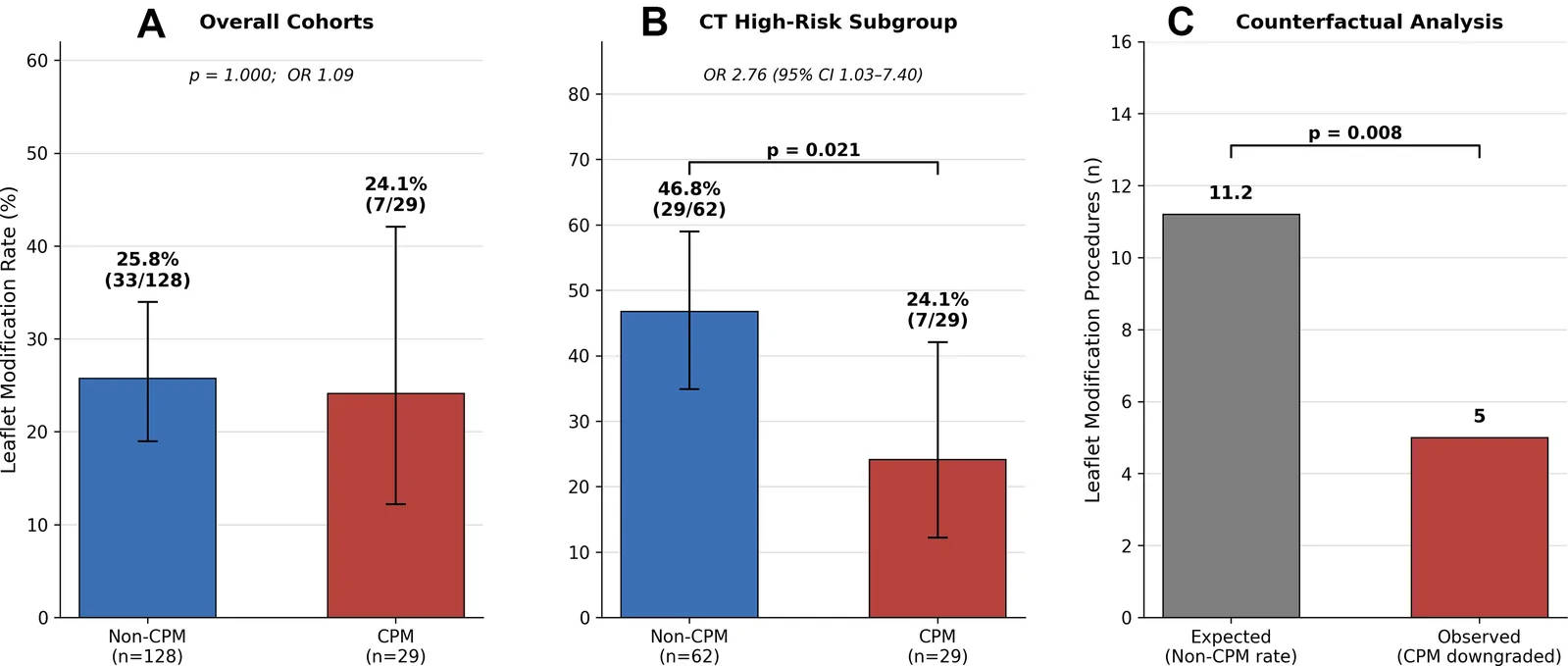
Reduced Leaflet Modification Rates Among High-Risk CPM-Augmented Patients (A) Comparison of leaflet modification procedures occurring in the overall non-CPM and CPM-augmented cohorts (25.8% vs 24.1%, respectively; *P* = 1.000). (B) Comparison of leaflet modification rates among the CT high-risk subgroups of the non-CPM and CPM-augmented cohorts (46.8% vs 24.1%, respectively; OR: 2.76; 95% CI: 1.03-7.40; Boschloo’s exact one-sided *P* = 0.021). (C) Counterfactual analysis of CPM-downgraded patients: 11.2 leaflet modification procedures were expected; 5 were observed (1-sided binomial *P* = 0.008). Error bars are reported as 95% Wilson confidence intervals. Abbreviations as in Figure 2.

**Central Illustration:**
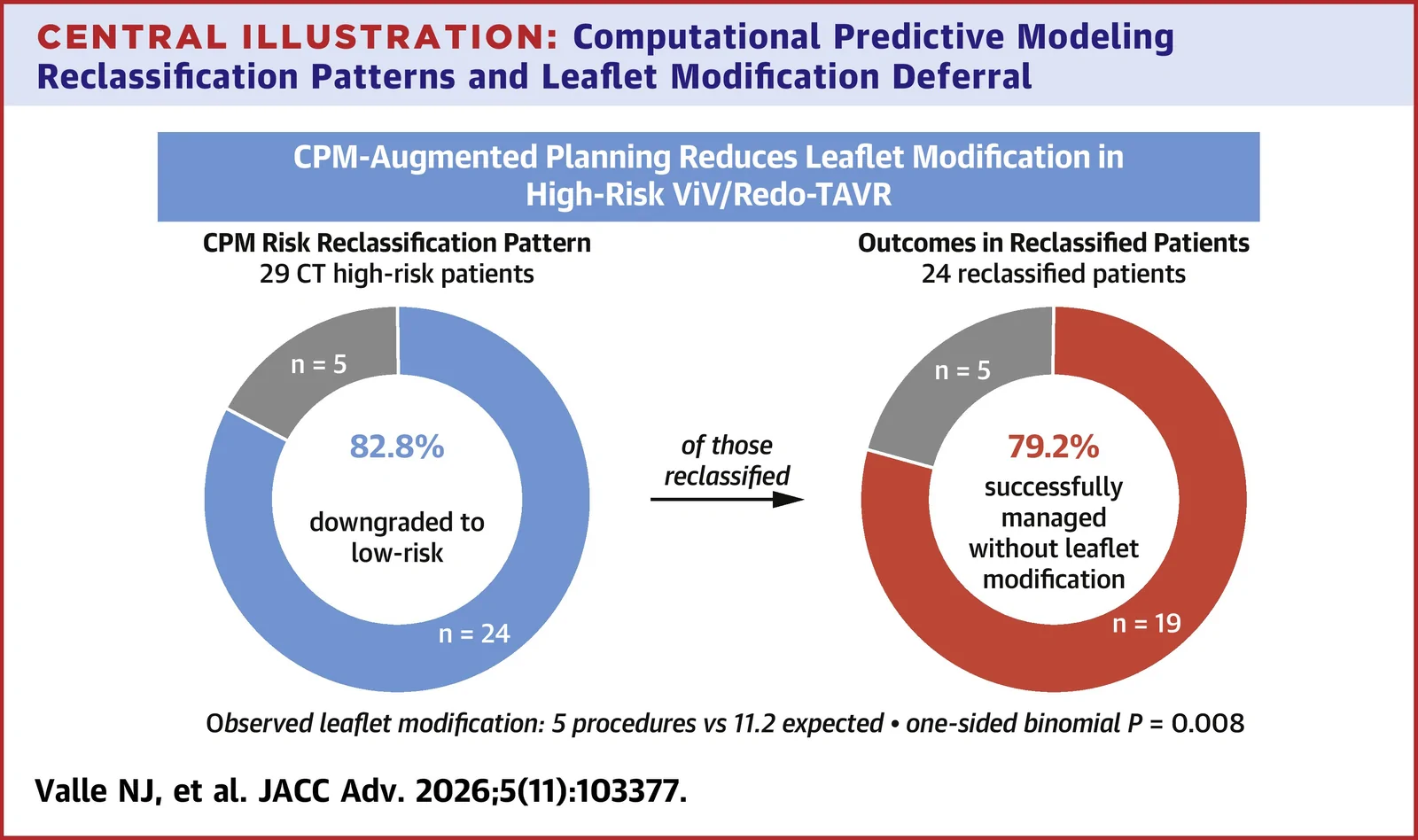
Computational Predictive Modeling Reclassification Patterns and Leaflet Modification Deferral (Left) Among 29 CT high-risk patients, CPM-augmented planning led to risk reclassification of 24 (82.8%) patients to low-risk status. (Right) Of the 24 risk-reclassified patients, 19 (79.2%) were successfully managed without leaflet modification. The observed leaflet modification rate of 20.8% was significantly lower than the expected rate of 46.8% (1-sided binomial *P* = 0.008). No coronary obstruction events were observed. CPM = computational predictive modeling; CT = computed tomography.

A counterfactual analysis was performed using an expected baseline LM rate derived from the high-risk non-CPM cohort (Figure 4C). Assuming this rate (46.8%), the expected number of LM procedures among the 24 risk reclassified patients was 11.2. Instead, 5 procedures were observed in the risk-downgraded group, suggesting that approximately 6 LM procedures were avoided in the CPM cohort (Supplemental Figure 1).

### Procedural resource utilization associated with LM

LM was associated with greater procedural resource utilization across the full cohort (Table 5, Figure 5). Patients receiving adjuvant LM procedures had significantly longer procedure times (91.3 ± 52.3 vs 52.0 ± 31.9 minutes; difference 39.3 minutes; *P* < 0.0001), greater contrast exposure (106.2 ± 87.5 vs 53.9 ± 37.0 mL; difference 52.3 mL; *P* < 0.0001), and longer fluoroscopy times (37.5 ± 23.5 vs 19.2 ± 14.5 minutes; difference 18.3 minutes; *P* < 0.0001) than patients who did not undergo LM. A direct comparison of procedure time, contrast exposure, and fluoroscopy time outcomes between the total CPM and non-CPM cohorts did not reveal significant differences (Table 6).

**Table 5:** Procedural Outcomes Associated With LM

	LM Performed (n = 41)	No LM (n = 117)	*P* Value
Procedure time, min	91.3 ± 52.3	52.0 ± 31.9	<0.001
Contrast exposure, mL	106.2 ± 87.5	53.9 ± 37.0	<0.001
Fluoroscopy time, min	37.5 ± 23.5	19.2 ± 14.5	<0.001

LM = leaflet modification.

**Figure 5:**
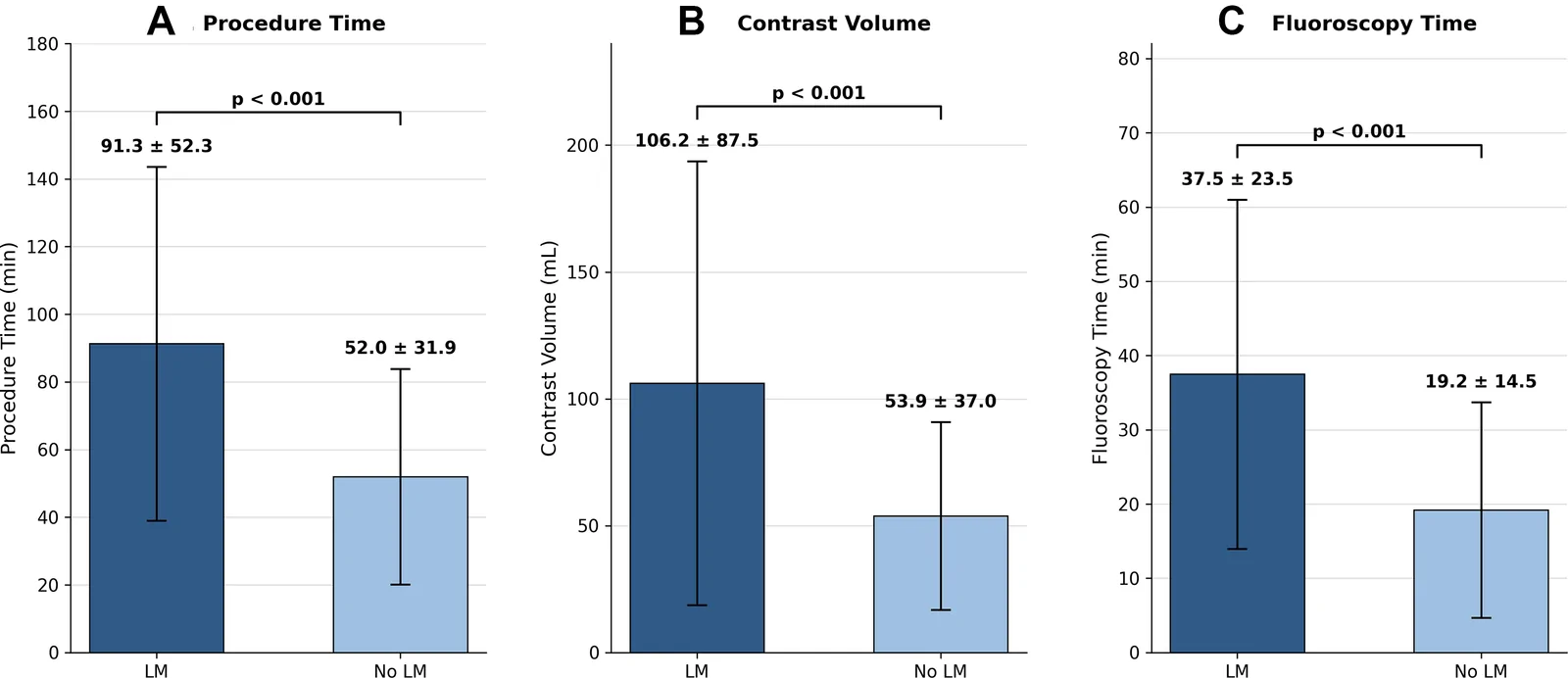
Procedural Resource Utilization Associated with Leaflet Modification (A) Procedure time, (B) contrast volume, and (C) fluoroscopy time were all significantly greater in patients who underwent leaflet modification (*P* < 0.001). LM = leaflet modification.

**Table 6:** Procedural Characteristics and In-Hospital Outcomes

	Non-CPM (n = 128)	CPM-Augmented (n = 29)	*P* Value
Transfemoral access, n (%)	125/128 (97.7%)	29/29 (100.0%)	1.000
Index replacement valve type			
Balloon-expandable valve, n (%)	38/128 (29.7%)	4/29 (13.8%)	0.104
Self-expanding valve, n (%)	90/128 (70.3%)	25/29 (86.2%)	
Leaflet modification, n (%)	33/128 (25.8%)	7/29 (24.1%)	1.000
Procedure time, min	62.9 ± 44.3	58.8 ± 30.0	0.554
Contrast exposure, mL	68.6 ± 62.8	60.7 ± 36.6	0.401
Fluoroscopy time, min	23.0 ± 18.1	28.2 ± 22.5	0.250
LOS, d	3.1 ± 4.1	3.5 ± 3.3	0.525
Post-TAVR AV mean gradient, mm Hg	8.5 ± 4.6	7.5 ± 4.4	0.258
In-hospital MACE, n (%)	6/128 (4.7%)	2/29 (6.9%)	0.641
In-hospital mortality, n (%)	3/128 (2.3%)	0/29 (0.0%)	1.000
Discharged alive, n (%)	125/128 (97.7%)	29/29 (100.0%)	1.000

Counts are stratified by utilization of CPM-augmented preprocedural planning.

LOS = length of stay; MACE = major adverse cardiac event; other abbreviations as in Table 1.

### Short-term clinical outcomes

In-hospital and short-term cardiovascular outcomes were comparable between the groups (Tables 6 and 7). In-hospital MACE occurred in 6 non-CPM patients (4.7%) and 2 CPM patients (6.9%; *P* = 0.64), and in-hospital mortality was 2.3% vs 0.0%, respectively (*P* = 1.00). Major or life-threatening bleeding occurred in 4 non-CPM patients (3.1%) and 0 CPM patients (*P* = 1.00). Cumulative all-cause mortality was 2.3% vs 0.0% at 30 days (*P* = 1.00), 4.7% vs 3.4% at 3 months (*P* = 1.00), and 10.2% vs 3.4% at 6 months (*P* = 0.47) for non-CPM and CPM patients, respectively. Follow-up transthoracic echocardiogram at 1 or 3 months is reported. There were no statistically significant differences in follow-up transthoracic echocardiogram between the non-CPM and CPM cohorts (52.2% vs 53.8%, respectively; *P* = 0.586). Three-point MACE was 6.2% vs 6.9% at 30 days (*P* = 1.00), 7.0% vs 10.3% at 3 months (*P* = 0.47), and 10.2% vs 10.3% at 6 months (*P* = 1.00) for non-CPM and CPM patients, respectively. Noncardiac causes of death were not captured. No significant differences in any short-term clinical outcomes were detected.

**Table 7:** Cumulative Clinical Outcomes Stratified by Utilization of CPM-Augmented Preprocedural Planning

Outcome	Non-CPM (n = 128)	CPM-Augmented (n = 29)	*P* Value
At 30 d			
MACE (composite), n (%)	8/128 (6.2%)	2/29 (6.9%)	1.000
Death, n (%)	3/128 (2.3%)	0/29 (0.0%)	1.000
MI, n (%)	0/128 (0.0%)	0/29 (0.0%)	1.000
Stroke, n (%)	5/128 (3.9%)	2/29 (6.9%)	0.614
At 3 mo			
MACE (composite), n (%)	9/128 (7.0%)	3/29 (10.3%)	0.465
Death, n (%)	6/128 (4.7%)	1/29 (3.4%)	1.000
MI, n (%)	0/128 (0.0%)	0/29 (0.0%)	1.000
Stroke, n (%)	6/128 (4.7%)	2/29 (6.9%)	0.641
At 6 mo			
MACE (composite), n (%)	13/128 (10.2%)	3/29 (10.3%)	1.000
Death, n (%)	13/128 (10.2%)	1/29 (3.4%)	0.469
MI, n (%)	0/128 (0.0%)	0/29 (0.0%)	1.000
Stroke, n (%)	9/128 (7.0%)	2/29 (6.9%)	1.000
Other outcomes			
Major/Life-threatening bleeding, n (%)	4/128 (3.1%)	0/29 (0.0%)	1.000
Follow-up left ventricular ejection fraction (TTE; 1 or 3 mo)	52.2 ± 14.0 (n = 122)	53.8 ± 12.9 (n = 24)	0.586

MI = myocardial infarction; TTE = transthoracic echocardiogram; other abbreviations as in Table 6.

## Discussion

### Principal findings

This study examined the real-world impact of CPM-augmented preprocedural planning on LM utilization in 157 consecutive patients undergoing ViV TAVR and redo-TAVR at a single high-volume center. Four principal findings emerge. First, adjunctive LM was performed in 26.3% of non-CPM ViV TAVR patients and 24.1% of non-CPM redo-TAVR patients, establishing LM utilization benchmarks for these populations that are largely absent from the existing literature. Second, among CT high-risk patients, the LM rate was significantly lower in patients who received CPM-augmented preprocedural planning (24.1% vs 46.8%; OR: 2.76; 95% CI: 1.03-7.40; *P* = 0.021); CO did not occur in either group. Third, CPM led to the reclassification of 82.8% of CPM-augmented patients to low-risk status, and the observed LM rate in reclassified patients (20.8%) was significantly lower than the reference rate (46.8%; *P* = 0.008). Lastly, each LM procedure was associated with an estimated 40 minutes of additional procedure time, 52 mL of additional contrast, and 18.3 minutes of additional fluoroscopy time when compared to patients who did not receive LM.

### LM utilization rates and procedural outcomes associated with LM

Despite growing ViV TAVR and redo-TAVR volume, population-level LM utilization data in exclusively ViV TAVR and redo-TAVR cohorts remain limited. In addition to filling this gap, our findings conceptualize the proportion of patients in these populations that are at risk of undergoing potentially unnecessary LM procedures. The main driver of LM procedures among our study population was CO risk determination based on static, 2D measurements derived from CTA.

In our study population, LM procedures were associated with several clinically relevant procedural outcomes. First, procedure time was increased by approximately 40 minutes on average in patients receiving adjunctive LM. This translates into approximately one additional TAVR that might have been performed in the time needed for each LM.^18^ The cost of better up-front procedural planning is likely offset by more efficient use of operative facility time.

In addition, each LM procedure was associated with approximately 52 mL of additional contrast exposure, a finding which has important renal implications given that 4% to 7% of ViV TAVR and redo-TAVR patients experience acute kidney injury after their procedure.^19^, ^20^ Moreover, chronic kidney disease patients requiring additional diagnostic or therapeutic procedures during index admission may be deferred or delayed if their initial procedural contrast exposure exceeds institutional thresholds.^21^ Lastly, contrast volume-to-creatinine clearance ratio and acute kidney injury after TAVR have both been independently linked with adverse cardiovascular outcomes.^22^, ^23^ These considerations emphasize the clinical significance of potentially reducing patient contrast exposure via more sophisticated preprocedural planning.

### Effects of CPM-augmented planning on LM rates

CTA-derived risk parameters (ie, CH, VTc, and VTstj) have guided CO risk stratification for nearly 2 decades but are derived from an operator-dependent process which does not necessarily account for patient-specific coronary ostial morphology.^15^, ^16^ We believe that the consequence is systematic overclassification of CO risk. For example, an analysis validating the VIVID classification conducted by Tomii et al^24^ found that among 96 ViV TAVR patients, 30 were classified as high risk using CTA. Coronary protection was performed in 3 of these, yet no CO events were observed in the other 27 patients.^24^ Similarly, Kitamura et al^12^ found that only 37.5% of lacerated leaflets in their cohort exhibited complete threatened CO on post-BASILICA CT, suggesting that up to 60% of LM procedures were unnecessary. The absence of CO events among our 19 risk-reclassified, LM-deferred patients provides further experience to this evidence base. Our counterfactual analysis suggests that 55.4% of expected LM procedures were avoided by CPM-augmented preprocedural planning (*P* = 0.008).

### Effects of CPM-augmented planning on physician decision-making

There is now a growing body of literature describing CPM-based assessment of pre-TAVR CTAs.^25^, ^26^, ^27^ However, these studies largely focus on the technical validation of various algorithms’ ability to obtain accurate and clinically relevant data from CT scans. Exploration of the way these technologies impact physician decision-making is still limited. Similar to the present study, Holst et al^14^ retrospectively analyzed the effects of CPM-augmented preprocedural modeling on LM rates in 116 TAVR patients and found that CPM was a useful enhancement to their preprocedural planning workflow. Our study expands on this work in several important ways. First, our population is exclusively patients with previously implanted AV bioprostheses, the highest-risk anatomical subgroup for CO. In addition, 45.7% of the Holst cohort underwent native TAVR procedures, potentially diluting the signal from the highest-risk TAVR subgroups. Lastly, we use a more sophisticated analytical framework which better captures the clinical ramifications of CPM-augmented preprocedural planning. Specifically, we found that CPM-augmented planning led to risk downgrading in 82.8% of patients considered high risk by conventional CTA-based methods. Ultimately, 79.2% of these downgraded patients were successfully managed without LM (n = 19).

The primary CPM-derived metric that contributed to LM decision-making was the DLC/d ratio. DLC/d is a metric that normalizes leaflet-to-coronary distance to the coronary ostial diameter, providing individualized CO risk evaluation that may be superior to traditional CTA-derived metrics.^14^, ^15^ In previous literature, DLC/d has been validated against a cohort of patients with a low coronary and/or small sinus (n = 28) including 6 of 28 cases that had confirmed CO.^16^ In the study, DLC values were validated through in vitro techniques with 3D printed models, and simulated DLC/d had a predictive power of 100% sensitivity and 95.7% specificity using a ratio of 0.7. Importantly, there is currently no literature defining gradation of CO risk with respect to various DLC/d values, and this represents an important area for future research. The safe and significant reduction in LM rates we observed among reclassified patients does provide some real-world clinical validation of DLC/d as a biomarker of CO risk in ViV TAVR and redo-TAVR. Prior literature has described this CPM model in ViV TAVR including the use of both balloon-expandable and self-expanding valves. The authors found that simulated models demonstrated reasonable agreement with actual post-CT scan measurements of the implanted THV in 2 selected cases.^28^ DLC/d across the study population was significantly different when compared with VTc, highlighting that the 2 measurements (and the information they provide about CO risk) are not equal. Importantly, this study was conducted in aortic root–replacement patients. Consistent with previously described TAVR CPM approaches, the platform described here reconstructs patient-specific antatomy and simulates valve deployment using a physics-based finite-element method rather than imaging-classifier artificial intelligence. Reported geometric accuracy from the platform’s 510(k) clearance was a simulated device-diameter error of ≤2 mm; this figure, importantly, derives from the trileaflet (native) AV anatomy (n = 89) and does not represent a CO misclassification rate or a ViV/redo-specific validation.^29^, ^30^ There is currently no literature that validates the CPM platform used in this study specifically for SEVs, aside from the clinical observational studies cited earlier. Further validation of this CPM platform in ViV TAVR procedures and in SEVs is needed.

### Study limitations

This study has several important limitations. The retrospective single-center design precludes causal inference, and unmeasured confounding is not excluded. CPM utilization was at operator and multidisciplinary heart team discretion. The anatomical differences between groups suggest that CPM patients represented a more anatomically complex subgroup within the CT high-risk cohort. It is possible that this may have independently influenced procedural decision-making. The sex imbalance between groups (62.1% female in CPM vs 35.9% in non-CPM subgroups; *P* = 0.01) is notable, as female sex is associated with AV anatomical differences that may independently influence risk classification and procedural outcomes. The numerically higher proportion of balloon-expandable valves observed in the non-CPM cohort likely reflects evolving institutional valve-selection practices over the study period and adoption of newer self-expanding models. The differences in CPM between balloon-expandable valves and SEVs warrant further study. The 29 patients that received CPM preprocedural planning augmentation were chosen by physicians, which introduces selection bias (though, in some ways, this bias is useful given that selection for CPM was largely based on CO risk). The absence of CO events in both groups is reassuring, though our study is underpowered to detect rare events. Importantly, it should be noted that CO can be caused by commissural misalignment, even when LM is performed successfully. Our center uses a high percentage of SEVs, which reduces the risk of commissural misalignment considerably. The utilization of CPM and similar technologies to augment decision-making with respect to this phenomenon, as well as in paravalvular leak prediction, is still largely unstudied and represents an important area for further study. The CPM model used in this study, to date, has FDA clearance for native TAVR only; however, the way its systems predict CO risk in ViV TAVR and redo-TAVR is conceptually identical and derived from anatomical reconstruction of the patient’s anatomy, with implantation of high-fidelity THV models. Prospective studies with larger sample sizes are required to formally validate the utilization of CPM to augment decisions surrounding LM in ViV TAVR and redo-TAVR. Generalizability is limited to high-volume centers with operators experienced in using CPM-integrated workflows. All findings should be considered exploratory and hypothesis-generating.

### Clinical implications and conclusions

The integration of CPM into preprocedural planning for ViV TAVR and redo-TAVR represents a promising refinement to a risk-stratification system that has remained largely static despite increasing TAVR volume and complexity. For the growing population of patients at elevated risk for CO, simulation-derived risk models provide individualized data that static CTA-derived measures cannot, and the findings presented here suggest that a meaningful proportion of LM procedures may be avoidable without compromising safety. Given the procedural complexity, harmful exposures, and operator expertise required for BASILICA and related techniques, safe deferral of LM carries significant clinical value. Multicenter prospective studies are needed to further evaluate the efficacy of CPM-augmented preprocedural planning in the safe deferral of LM procedures without increasing the incidence of CO.

## Funding support and author disclosures

Dr Benza has received speaking, consulting, and proctoring income from Abbott. Dr Kemp was a consultant and proctor for Medtronic and Abbot structural heart. Dr Talreja has received speaking, consulting, and proctoring income from Medtronic, Abbott, Abiomed, Bristol Myers Squibb, Novartis, and Amgen. Dr Peterson has received research funding from Edwards Lifesciences and consulting and speaking income from Egnite Inc, Edwards Lifesciences, Medtronic, Amgen, Xenter, and CRF. Dr Mahoney has received consulting and proctoring income from Abbott, Medtronic, and Edwards. Dr Cohen has received consulting income from and served as educational faculty for Medtronic, Phillips, and Abbott. Dr Summers has received speaking, consulting, and proctoring income from Abbott and Medtronic, and he served on the advisory board for Abbott, Medtronic, Picardia, Shockwave, DASI Simulations LLC, and Abiomed. All other authors have reported that they have no relationships relevant to the contents of this paper to disclose.
