# Supplementary material for: Computational Modeling Reduces Leaflet Modification Rates in Valve-In-Valve and Redo Transcatheter Aortic Valve Replacement
Source: JACC Adv. 2026 Oct 23;5(11):103377. doi: 10.1016/j.jacadv.2026.103377 (PMC13721089; doi:10.1016/j.jacadv.2026.103377)
Supplement: Supplementary file 1 [file mmc1.docx]

# **Supplemental Methods Table 1. Adoption of PRIME 2.0 Checklist for Novel Computational Predictive Modeling Technologies**

| **PRIME 2.0 domain / item** | **Checklist Expectation** | **Reporting for the Present Study** | **Source / Status** |
| --- | --- | --- | --- |
| 1.1 Appropriateness of AI/Computational Predictive Modeling | Describe the need for, and appropriateness of, applying the method. | CPM was used to refine coronary-obstruction (CO) risk assessment beyond static 2-D CTA measures (coronary height, VTc, VTstj) in the highest-risk anatomic subgroup (ViV/redo-TAVR). Rationale is detailed in the main-text Introduction and Methods. | Disclosed (main text; refs 14–16) |
| 1.2 Objectives, input data, prediction target | AI task and deployment context; input data and number of training/test examples; supervision type; nature of output. | Task/deployment: patient-specific simulation of post-deployment transcatheter heart valve (THV) geometry to derive interpretable CO-risk markers used at pre-procedural heart-team planning.  Input: patient pre-procedural cardiac CTA.  Output: a continuous geometric measurement (DLC; DLC/d), thresholded at ≤0.7 for high risk. This is not a black-box class label.  Supervision type / training–test counts: the platform is a physics-based computational simulation, not a supervised classifier; the developer states it does not use traditional medical-imaging AI architectures. Training/validation datasets are proprietary (shared with the FDA but not public). | Partly Disclosed (workflow/output); training data Proprietary / not disclosed |
| 1.3 Design of the AI study | Study design; data origin; whether impact analysis was included. | The present study is a single-center retrospective real-world evaluation of the platform's clinical impact on leaflet-modification (LM) rates. Importantly, the present study is not a model-development study. Data origin: one high-volume U.S. structural-heart center. Impact analysis: LM-rate reduction and counterfactual estimate (main text). | Disclosed (main text) |
| 2.1 Data format | Technical details of data acquisition and data format. | Input was standard institutional pre-procedural cardiac CTA. Acquisition parameters were not standardized across this retrospective cohort and are not separately tabulated; platform-side ingestion/format requirements were not provided to investigators. | Partly Disclosed / Proprietary / not disclosed |
| 2.2 Clinical characteristics of the cohort | Age, sex, race/ethnicity; key clinical, treatment and imaging characteristics; cases vs controls. | Reported in main-text Table 1, including documented between-group imbalances (female sex, aortic annular area and perimeter, annular calcification) carried forward as limitations. | Disclosed (Table 1) |
| 2.3 Steps of data preprocessing | Cleaning/harmonization; missing values; outliers; class imbalance. | Anatomic reconstruction and segmentation were performed off-site by the developer using in-house segmentation tools (stated by the developer to accommodate all clinically relevant aortic-valve morphologies); investigator-side preprocessing was limited to standard clinical CTA handling. Internal harmonization, missing-data and outlier handling are not disclosed. | Per the Developer (in-house segmentation) / Proprietary / not disclosed (internal steps) |
| 2.4 Feature engineering / selection | Feature-engineering and feature-selection techniques. | The clinically actionable features are DLC (virtual THV leaflet-to-coronary-ostium distance) and DLC/d (indexed to ostial diameter). Ostial diameter (d) is taken directly from the patient CTA; DLC is measured from the simulated THV leaflet to the CTA-derived reconstruction. Any internal representations are not disclosed. | Disclosed (markers; refs 15,16) / Proprietary / not disclosed (internals) |
| 3.1 Data composition | Define structured vs unstructured data composition. | Structured geometric/anatomic data derived from a single imaging modality (CTA). No natural-language or multimodal text inputs. | Disclosed |
| 3.2–3.4 Training strategies / method | AI method/application used, with rationale for clinical-task fit. | Per the developers, the platform performs in-house anatomic reconstruction and an in-house finite-element, physics-driven simulation that couples finite-element techniques with known TAVR boundary conditions, tuned and validated against post-CT scans, to simulate balloon- and self-expanding valve deployment and derive geometric markers. Per the developer, the simulation platform does not use traditional medical-imaging AI architectures; the approach follows prior published TAVR computational-simulation methods (refs 16, 29, 30). One developer-authored paper (ref 27) labels a related workflow a “3-D artificial-intelligence algorithm.” Solver settings, boundary-condition specifics and parameters are proprietary. The exact nature of if and how the simulation utilizes “AI” and/or “algorithmic approaches” is proprietary. | Developer response received (method class) / Proprietary / not disclosed (parameters/nature of AI utilization/nature of algorithmic approaches) |
| 3.5 Solving clinical problems (segmentation / multi-source) | For segmentation, architecture and temporal handling; for multi-source, integration method. | Segmentation uses in-house tools (per the developer); the specific method and any temporal handling are not disclosed. This is a single-modality (CT) application with no multi-source data fusion and no NLP/report-generation component. | Developer response received (in-house) / Proprietary / not disclosed (method); Not applicable (fusion/NLP) |
| 4.1 Importance of model assessment | Evaluation approach and how it addresses the clinical question. | In this study the evaluation target is clinical impact (LM deferral and CO events), not standalone model metrics (i.e. directly addressing whether CPM safely reduces unnecessary LM. | Disclosed (main text) |
| 4.2 Technical performance metrics | Report and justify performance metrics; annotation process, reference-standard prep, observer variability. | Developer-provided geometric-accuracy figure: the PrecisionTAVI 510(k) summary (K223809) reports a simulated device-diameter error of ≤2 mm in trileaflet (“tricuspid”) aortic-valve patients (n=89; a native-anatomy geometric metric, *not* a CO-risk misclassification rate and not a ViV/redo cohort. Independently, the DLC/d marker at a 0.7 threshold showed 100% sensitivity / 95.7% specificity in a native low-coronary/small-sinus cohort (n=28; 6 CO) with in-vitro 3-D-printed validation (ref 16). Ref 28 reports only qualitative geometric agreement (n=2). The platform's own CO-risk misclassification rate and observer-variability data were not disclosed. | Developer response received (510k diameter error); Ref 16 (marker); Ref 28 (n=2) / Proprietary / not disclosed (CO-risk error rate) |
| 4.3–4.4 Robustness / generalizability | Robustness to external variation; performance across subpopulations. | The developer maintains that FDA verification/validation addressed sample size and morphological, geographic, racial and ethnic representation, but that these data are proprietary. Reported accuracy (510k; ref 16) is native/trileaflet anatomy; no published ViV/redo- or self-expanding-valve-specific validation exists. Regulatory clearance is for native TAVR only. | Disclosed (limitations) / Proprietary / not disclosed (V&V data) |
| 4.5 Interpretability / uncertainty | Interpretability/explainability methods; epistemic and aleatoric uncertainty. | Outputs are inherently interpretable radiologic markers (DLC, DLC/d) plus color-coded 3-D reconstructions (Figure 1) interpreted and contextualized by a multidisciplinary heart team, not an isolated score. No formal per-case uncertainty quantification is provided, and there is no published gradation of CO risk across DLC/d values (marker used dichotomously at 0.7). | Disclosed (interpretable markers) / Not Provided (uncertainty) |
| 5.1 Misclassification impact | Potential clinical impact of misclassification. | Addressed in main text: a false “high-risk” output risks unnecessary LM (added time, contrast, fluoroscopy); a false “low-risk” output carries a theoretical CO risk. No CO events occurred in either study group. | Disclosed (main text) |
| 5.2 Clinical-utility metrics | Clinical utility; cost-effectiveness / resource use. | LM deferral and procedural resource use (procedure time, contrast, fluoroscopy) are reported in main text. A formal cost-effectiveness analysis was not performed. | Disclosed / not performed (formal CEA) |
| 5.3 Clinical validation | Evidence of clinical validation. | Prior clinical evidence: integration of the platform into TAVR planning reported 100% effectiveness in avoiding CO (n=116; ref 14). The present study adds exploratory real-world evidence for DLC/d-augmented planning in ViV/redo TAVR; there is no prospective or randomized validation in this setting. | Disclosed (ref 14; present study, exploratory) |
| 5.4 Continuous monitoring | Plans for post-deployment monitoring. | Not applicable to this retrospective study; platform-level post-market monitoring is a developer/regulatory function outside the present scope. | Not applicable |
| 6.1 Transparency / FAIR | Data sharing per FAIR; training-data representativeness. | The model-generation algorithm and training/validation data are proprietary and were not shared; the developer regards V&V data shared with the FDA as outside the scope of an author (user) publication. Study data are governed by IRB terms. | Proprietary / not disclosed (algorithm/data) / Disclosed (limits) |
| 6.2 Technical reproducibility | Final model + training process; randomness; seeds; hardware; justify if code/weights/data not shared. | Platform internals (code, parameters, hardware) were not shared because the platform is a commercial, FDA-cleared product to which investigators had no access (an explicit, justified non-disclosure). The clinical workflow is reproducible (CTA → developer-generated report → heart-team review). | Proprietary / not disclosed (justified non-disclosure) |
| 6.3 LLM / generative-AI specifics | Model name/version/provider; prompts; usage context; output variability. | Per the developer the simulation does not use traditional AI architectures that are commonly used in medical imaging. | Proprietary / not disclosed |
| 7.1 Study / model limitations | Key limitations; sensitivity analyses. | Enumerated in main-text Limitations. | Disclosed |
| 7.2 Study strengths | Articulate strengths. | Human-interpretable markers; multidisciplinary heart-team adjudication; consecutive real-world cohort; transparent scoping of proprietary limits. | Disclosed |
| 7.3 Bias / fairness | Stratified performance by subgroup; mitigation; fairness limits. | Between-group imbalances (sex, annular size, calcification) are reported (Table 1) and selection bias is acknowledged. Developer states FDA V&V considered demographic and morphological representation, but subgroup performance data are not public. | Disclosed (study) / Proprietary / not disclosed (platform subgroup data) |
| 7.4 Alternative methods | Benchmark vs standards; justify complexity; complementary methods. | Benchmarked against conventional CTA metrics (CH, VTc, VTstj) and prior literature (e.g., VIVID); the DLC/d-vs-VTc discordance is highlighted (ref 28). Complementary non-CPM approaches are discussed in main text. | Disclosed (main text; refs 24,28) |

^1^AI = artificial intelligence; CPM = computational predictive modeling; CO = coronary obstruction; CTA = computed tomography angiography; VTc = valve-to-coronary distance; VTstj = valve-to-sinotubular junction-distance; ViV = valve-invalve; TAVR = transcatheter aortic valve replacement; THV = transcatheter heart valve; DLC = distance from leaflet to coronary ostium; d = coronary diameter; LM = leaflet modification; NLP = natural language processing; V & V = verification and validation; IRB = institutional review board; CH = coronary height.

^2^This supplement describes the computational predictive modeling (CPM) approach used in the parent study, structured to the domains of the PRIME 2.0 checklist for cardiovascular imaging–related Artificial Intelligence (Kagiyama, Tokodi, Hathaway et al; *JACC Cardiovasc Imaging* 2026;19[2]:225–251), with particular reference to its Table 2. The parent study is a clinical-impact evaluation of a commercially available platform (PrecisionTAVI; DASI Simulations, LLC) by a user (author) group, not a model-development report.

^3^The investigators had no role in developing the platform and no access to its internal algorithm or its verification/validation datasets, which the developer holds as proprietary (shared with the FDA and not public). To keep this account honest and verifiable, each item is tagged: **Disclosed** (supported by the parent manuscript, the clinical workflow, or peer-reviewed literature), **Developer provided/Per developer** (provided by the developer), or **Proprietary / not disclosed** (requested but not provided). Where an item is proprietary, it is stated plainly in the table.

^4^Per the developer, the platform does not use traditional medical-imaging AI architectures; it is a physics-based computational simulation (in-house anatomic segmentation and an in-house finite-element model coupling finite-element techniques with known TAVR boundary conditions, tuned and validated against post-implant CT). We map it to PRIME 2.0 because it is a common framework for novel computational methods, not because it is a conventional imaging classifier.

**Supplemental Figure S1.**
